# Comparison of 3-month visual outcomes of a new multifocal intraocular lens vs a trifocal intraocular lens

**DOI:** 10.1097/j.jcrs.0000000000000971

**Published:** 2022-05-12

**Authors:** H. Burkhard Dick, Robert E. Ang, Dean Corbett, Peter Hoffmann, Manfred Tetz, Alberto Villarrubia, Carlos Palomino, Alfredo Castillo-Gomez, Linda Tsai, Eugenia K. Thomas, Priya Janakiraman

**Affiliations:** From the University Eye Hospital, Ruhr University, Bochum, Germany (Dick); Asian Eye Institute, Makati City, Philippines (Ang); Auckland Eye Limited, Auckland, New Zealand (Corbett); Augen- & Laserklinik, Castrop-Rauxel, Germany (Hoffmann); Augentagesklinik am Spreebogen, Berlin, Germany (Tetz); Hospital La Arruzafa, Córdoba, Spain (Villarrubia); Hospital Universitario Quirónsalud Madrid, Madrid, Spain (Palomino, Castillo-Gomez); Johnson & Johnson Vision, Santa Ana, California (Tsai, Thomas, Janakiraman).

## Abstract

To compare the clinical performance of the TECNIS Synergy multifocal (model ZFR00V) intraocular lens (IOL) with that of the AcrySof PanOptix Trifocal (model TFNT00) IOL in patients undergoing bilateral cataract surgery.

**Setting:**

Multicenter clinical setting.

**Design:**

Prospective randomized comparative study.

**Methods:**

Patients aged 22 years or older were randomly assigned (2:1) to bilateral implantation with ZFR00V or TFNT00 IOLs. End points included the mean binocular distance-corrected near visual acuity (DCNVA) at 40 cm, photopic and mesopic DCNVAs at 33 cm, photopic low-contrast corrected distance visual acuity (CDVA) and mesopic CDVA, nondirected patient responses to an ocular/visual symptoms questionnaire, and safety.

**Results:**

Of the 150 patients implanted with IOLs, 95 of the 97 patients with ZFR00V IOLs and 52 of the 53 patients with TFNT00 IOLs completed the 3-month follow-up. Most patients in the ZFR00V and TFNT00 groups achieved 20/25 or better binocular CDVA (100% vs 96.2%) and DCNVA measured at 40 cm (88.4% vs 75.0%) and 33 cm (78.9% vs 51.9%). The mean between-group difference in binocular DCNVA at 40 cm favored ZFR00V IOLs (0.5 lines Snellen; 95% CI, 0.012 to 0.089; *P* ≤ .05). Similarly, the mean binocular photopic and mesopic DCNVAs at 33 cm (0.8 lines Snellen each; both *P* ≤ .05 vs TFNT00) and photopic high-contrast and low-contrast CDVA (0.5 lines Snellen each; both *P* ≤ .05 vs TFNT00) favored ZFR00V IOLs. Patient-reported ocular/visual symptoms and safety were generally similar between the 2 IOLs.

**Conclusions:**

The ZFR00V IOL showed an extensive range of vision, particularly through near distances, and better mesopic performance than the TFNT00 IOL in patients undergoing cataract surgery.

## Abstract 2

TECNIS Synergy multifocal intraocular lens, model ZFR00V, showed improved clinical performance particularly through near distances vs Acrysof PanOptix Trifocal intraocular lens, model TFNT00, in patients undergoing bilateral cataract surgery.

New intraocular lenses (IOLs) that bridge the gap between the performance of monofocal and multifocal IOLs include the TECNIS Symfony extended-range-of-vision IOL and the AcrySof PanOptix Trifocal IOL, model TFNT00 (Alcon Laboratories, Inc.).^1^ In addition, the new TECNIS Synergy IOL, model ZFR00V, combined the diffractive technologies derived from multifocal and Symfony extended-range-of-vision IOLs to provide good distance visual acuity with a continuous range of high-quality vision through intermediate and near distances. This study aimed to evaluate the clinical performance of the TECNIS Synergy IOL, model ZFR00V, vs the AcrySof PanOptix Trifocal IOL, model TFNT00 and to present key results at the postoperative 3-month timepoint.

## METHODS

### Study Design

This prospective, bilateral, randomized, comparative study was conducted across 12 sites in Germany, Spain, Philippines, New Zealand, and Singapore (German Clinical Trials Register, DRKS00016732). However, COVID-19 pandemic restrictions limited the availability of final data from 1 study site; data from this site were not included in this analysis.

The study was approved by the Institutional Review Board/Independent Ethics Committee at each study center and was conducted in accordance with Good Clinical Practice guidelines (CPMP/ICH/195/35), ISO14155:2011, tenets of the Declaration of Helsinki, and all other applicable laws and regulations of the participating countries. All patients provided written informed consent before participating in the study.

### Patients

Patients aged 22 years or older with planned bilateral cataract or clear lens extraction (3-4 cases in each IOL group) and posterior chamber IOL implantation were included. Patients were excluded if they required IOL powers outside the range of +14.0 to +26.0 diopters (D); all other exclusion criteria were intended to limit confounding factors. See Supplemental Digital Content, Methods (http://links.lww.com/JRS/A598), for detailed inclusion/exclusion criteria.

### IOL Description

The TECNIS Synergy OptiBlue IOL, model ZFR00V (power: +5.0 to +34.0 D in 0.5 D increments), has a proprietary diffractive surface derived from a combination of extended depth-of-focus and multifocal technologies and is designed to correct chromatic aberration and provide a range of vision from distance to near.^2^ In addition, the Synergy IOL includes violet light–filtering chromophore, which reduces transmittance of violet light wavelengths.^2^ The AcrySof PanOptix Trifocal IOL, model TFNT00 (power: +6.0 to +30.0 D in 0.5 D increments; +31.0 to +34.0 D in 1.0 D increments), has a biconvex optic containing an aspheric design and diffractive structure on the anterior surface.^3^ The diffractive structure divides incoming light to provide a range of vision from distance to near.^3^

### Procedures and Assessments

**Figure 1.**
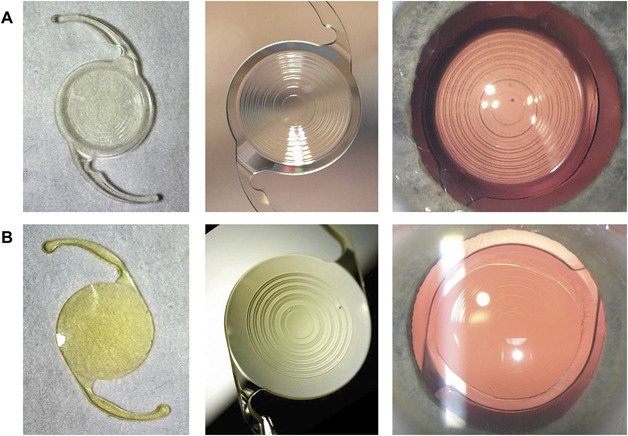
Photographs of (*A*) TECNIS Synergy IOL, model ZFR00V, and (*B*) AcrySof IQ PanOptix Trifocal IOL, model TFNT00.

Patients were randomly assigned (2:1) using an electronic data capture system (Merge eClinical OS) to undergo implantation with either the ZFR00V or TFNT00 IOLs in both eyes (Figure 1). Lens power calculations were completed prior to randomization. All patients and technicians remained masked to the implanted IOLs throughout the study. Intraoperatively, each patient was issued a temporary IOL implant identification card that excluded details on the implanted IOLs for masking. The temporary card was replaced with a permanent card identifying the implanted IOLs after the final study visit.

Each surgeon used their standard, small-incision, phacoemulsification cataract extraction technique to implant study IOLs into the capsular bag. Medications were used as needed by each investigator, and no refractive procedures were performed during the study. All patients underwent a preoperative examination within 60 days of the first surgery; postoperative examination ≤30 days between the first-eye and second-eye surgeries; and postoperative examination of both eyes at 3 months (60 to 90 days after the second-eye surgery).

Visual acuity was measured using the Early Treatment of Diabetic Retinopathy Study (ETDRS) chart in the Clinical Trial Suite (CTS; M&S Technologies, Inc.), and the total number of letters read were recorded and converted to logMAR for analyses. Distance measurements for binocular corrected distance visual acuity (CDVA) were tested at 4.0 m under 100% contrast photopic (approximately 85 cd/m^2^) and mesopic conditions (approximately 3 cd/m^2^) and under low-contrast (25%) photopic conditions.^4^ All binocular distance-corrected near visual acuities (DCNVAs) were tested at 40 cm and 33 cm under photopic conditions and at 33 cm under mesopic conditions. Binocular distance-corrected intermediate visual acuity (DCIVA; +0.25 D to ETDRS sphere only) was tested under photopic lighting conditions at 66 cm.^5^ Manifest refractions were performed using the maximum plus refraction technique in the CTS at a distance of 4.0 m. For refractive outcomes, raw data were converted to plus cylinder format and adjusted for optical infinity (−0.25 D of sphere). Combined visual acuity was determined by calculating the proportion of patients with a given acuity level at all measured distances. Defocus testing was performed under photopic conditions using the CTS at 4.0 m with the ETDRS refraction in place (no adjustment necessary for test distance). Patients were defocused in −0.50 D increments from +2.00 D to −4.00 D, and the number of letters read correctly at each defocus increment was recorded. The defocus diopters at which 0.2 logMAR or better was achieved was determined by visual inspection.

### End Points

The study end points included the assessment of refractive outcomes, binocular visual acuity, distance-corrected defocus testing, subjective outcomes, and safety at 3 months. For detailed end points, see Supplemental Digital Content, Methods (http://links.lww.com/JRS/A598).

### Statistical Analysis

The study aimed to enroll approximately 115 and 55 patients in the ZFR00V and TFNT00 IOL groups, respectively (for detailed sample size calculations, see Supplemental Digital Content, Methods, http://links.lww.com/JRS/A598). SAS v. 9.4 was used for this analysis. All analyses included data from all eyes implanted with either ZFR00V or TFNT00 IOLs and with data available during analyses (ie, no data imputation). Summary statistics included sample size, mean, SD, and 2-sided 95% CI of the mean value as appropriate for continuous variables and the frequency and proportion of patients for categorical data. Two-sided testing with an alpha level of .05 was used for comparison between the IOL groups. The null hypothesis was that there was no difference between the IOL groups, and the alternative hypothesis was that there was a difference between the IOL groups. For continuous variables, *P* values of ≤.05 or >.05 were reported based on whether the 2-sided 95% CI of the difference between the groups overlapped with zero (ie, an identical analysis to a 2-sample 2-sided *t* test with an alpha level of .05). For categorical variables, 2-sided Fisher exact test was used for *P* value determination. For binocular defocus data, the mean visual acuity at each diopter was plotted by the IOL group.

## RESULTS

This analysis included 150 patients who were enrolled between June 25, 2019, and October 29, 2020, and bilaterally implanted with ZFR00V (n = 97) or TFNT00 (n = 53) IOLs. At the 3-month postoperative timepoint, data were available for 95 (97.9%) and 52 (98.1%) patients in the ZFR00V and TFNT00 IOL groups, respectively. Patient accountability in both IOL groups at the 3-month postoperative visit was >98% (Supplemental Digital Content, Table 1, http://links.lww.com/JRS/A598).

Baseline characteristics were comparable between the 2 groups and were not statistically different (*P* > .05) (Table 1). The mean age was 63.8 ± 8.0 years in the ZFR00V IOL group and 65.6 ± 7.8 years in the TFNT00 trifocal IOL group, with >50% female participants in both groups. Most of the patients in the 2 treatment groups were White (≥95%).

**Table 1.** Patient demographics

Characteristic	TECNIS Synergy (n = 100)	AcrySof PanOptix (n = 53)
Age, y		
Mean ± SD	63.8 ± 8.0	65.6 ± 7.8
Median (range)	62 (48, 82)	66 (51, 82)
Sex, n (%)		
Female	57 (57.0)	31 (58.5)
Male	43 (43.0)	22 (41.5)
Race, n (%)		
White	95 (95.0)	51 (96.2)
Asian	4 (4.0)	2 (3.8)
Black	0	0
Other	1 (1.0)	0
Ethnicity, n (%)		
Hispanic/Latino	47 (47.0)	23 (43.4)
Not Hispanic/Latino	53 (53.0)	30 (56.6)
Iris color		
Blue/gray	32 (32.0)	24 (45.3)
Brown/black	48 (48.0)	21 (39.6)
Green/hazel	20 (20.0)	8 (15.1)

The between-group difference for all parameters was not statistically significant (*P* > .05)

### Objective Outcomes

#### Refractive Outcomes at 3 Months

The mean refractive sphere, refractive cylinder, and manifest refraction spherical equivalent outcomes were within ±0.50 D for both IOL groups in each of the implanted eyes (Supplemental Digital Content, Figure 1, http://links.lww.com/JRS/A598). A similar proportion of patients in each group had manifest refraction spherical equivalent and absolute refractive cylinder within ±0.50 D and ±1.00 D in the first and second implanted eyes (Supplemental Digital Content, Table 2, http://links.lww.com/JRS/A598).

#### Corrected Visual Acuity Outcomes at 3 Months

**Figure 2.**
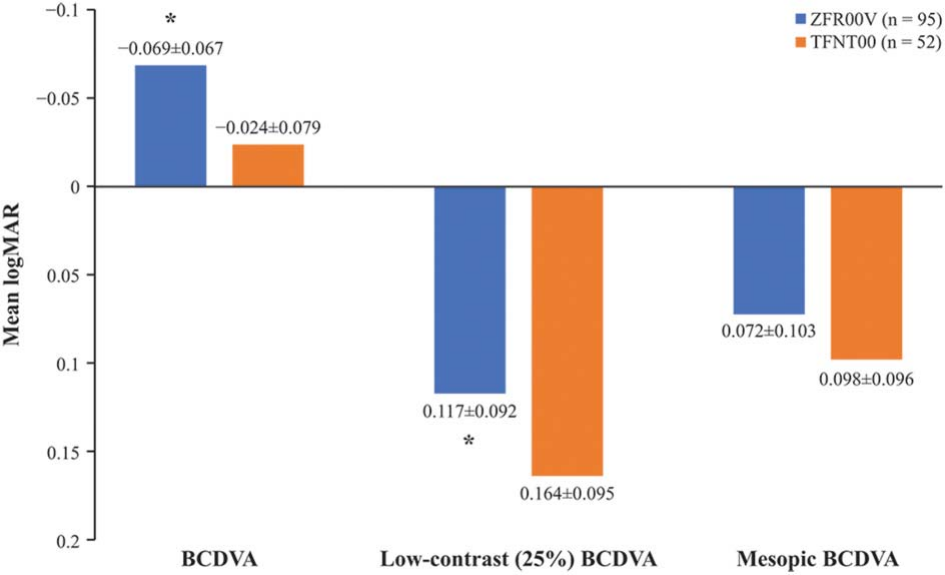
Binocular CDVA outcomes at 3 months postoperatively for the ZFR00V (n = 95) and (n = 52) TFNT00 IOLs. Values presented as mean ± SD; *P ≤ .05 vs TFNT00.

**Figure 3.**
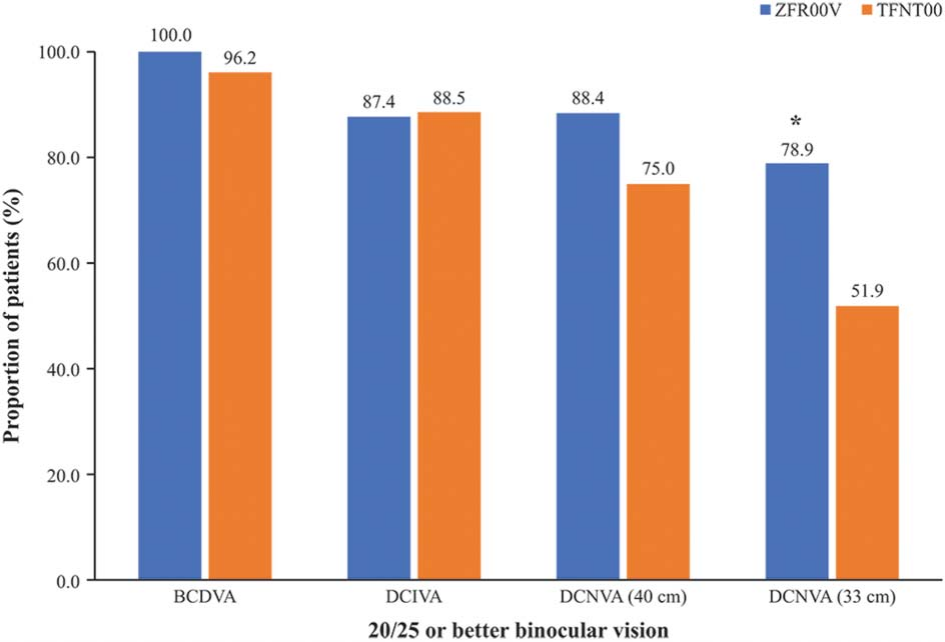
Proportion of patients who achieved 20/25 (0.8 decimal) or better binocular CDVA, DCIVA, and DCNVA at 3 months. *P ≤ .05 vs TFNT00 IOLs.

The mean (±SD) binocular CDVA was −0.069 ± 0.067 logMAR (Snellen 20/17; range: −0.26, 0.10) for ZFR00V IOL-implanted eyes and −0.024 ± 0.079 logMAR (Snellen 20/19; range: −0.20, 0.20) for TFNT00 IOL-implanted eyes, with a mean difference between lens groups of 0.5 lines (2.5 letters; 95% CI, 0.021 to 0.070; *P* ≤ .05), which favored the ZFR00V group (Figure 2). Most patients in the ZFR00V and TFNT00 groups achieved 20/25 or better binocular CDVA (95 [100%] and 50 [96.2%], respectively) (Figure 3). Under low-contrast (25%) photopic conditions, the difference between the IOL groups in the mean binocular CDVA was 0.047 logMAR (0.5 lines in Snellen; 95% CI, 0.016 to 0.079; *P* ≤ .05) in favor of the ZFR00V IOL (Figure 2). Low-contrast, photopic, binocular CDVA of 20/40 or better was achieved by 94 (98.9%) and 49 (94.2%) patients in the ZFR00V and TFNT00 IOL groups, respectively. Under mesopic conditions, the difference between the IOL groups in the mean binocular CDVA was 0.026 logMAR (0.3 lines Snellen equivalent; 95% CI, −0.008 to 0.060); this difference was not statistically significant (Figure 2). The binocular mesopic CDVA of 20/40 or better was achieved by 95 (100%) and 50 (96.2%) patients in the ZFR00V and TFNT00 IOL groups, respectively.

The mean binocular DCIVA was 0.012 ± 0.107 logMAR (Snellen 20/21; range: −0.20, 0.30) for the ZFR00V IOL group and 0.029 ± 0.135 logMAR (Snellen 20/21; range: −0.18, 0.66) for the TFNT00 IOL group, indicating similar performance between the 2 IOLs. Most patients in both IOL groups achieved 20/25 or better DCIVA (83 [87.4%] and 46 [88.5%], respectively) (Figure 3).

The mean binocular photopic DCNVA at 40 cm was 0.025 ± 0.112 logMAR (Snellen 20/21; range: −0.22, 0.36) for ZFR00V IOL-implanted eyes and 0.075 ± 0.114 logMAR (Snellen 20/24; range: −0.10, 0.36) for TFNT00 IOL-implanted eyes; the mean between-group difference was 0.5 lines (2.5 letters), favoring the ZFR00V IOL (95% CI, 0.012 to 0.089; *P* ≤ .05). A similar proportion of patients in the 2 IOL groups achieved binocular DCNVA of 20/32 or better at 40 cm (91 [95.8%] for ZFR00V vs 48 [92.3%] for TFNT00 IOLs). The proportion of patients who achieved 20/20 or better binocular photopic DCNVA at 40 cm was higher in the ZFR00V group (n = 59; 62.1%) than in the TFNT00 IOL group (n = 26; 50.0%).

At a closer distance of 33 cm, there was a larger difference observed between the 2 IOLs. The mean binocular photopic DCNVA was 0.072 ± 0.097 logMAR (Snellen 20/24; range: −0.12, 0.36) for the ZFR00V IOL and 0.149 ± 0.107 logMAR (Snellen 20/28; range: −0.06, 0.36) for the TFNT00 IOL; the mean between-group difference was 0.8 lines (4 letters; 95% CI, 0.043 to 0.111), favoring ZFR00V (*P* ≤ .05). A significantly higher proportion of patients in the ZFR00V group achieved binocular photopic DCNVA of 20/32 or better at 33 cm (93 [97.9%] vs 41 [78.8%]; *P* ≤ .05) and 20/25 or better photopic DCNVA measured at 33 cm (75 [78.9%] vs 27 [51.9%], respectively, *P* ≤ .05) vs the TFNT00 group (Figure 3). Under mesopic conditions, the difference between the IOL groups in the mean binocular DCNVA measured at 33 cm was 0.078 logMAR (0.8 lines Snellen; 95% CI, 0.039 to 0.117; *P* ≤ .05), favoring ZFR00V IOLs. The proportion of patients who achieved binocular DCNVA at 33 cm of 20/32 or better under mesopic conditions was significantly higher for the ZFR00V group than that of the TFNT00 IOL group (56 [58.9%] vs 15 [28.8%], respectively; *P* ≤ .05).

The analysis of combined visual acuity included all patients who achieved 20/25 or better visual acuity at all measured distances (far, intermediate, and near). For combined visual acuity, a significantly higher proportion of patients with ZFR00V IOLs achieved 20/25 or better binocular corrected visual acuity at all distances tested, from far to near (33cm), compared with patients with TFNT00 IOLs (70 [73.7%] vs 26 [50%]; *P* ≤ .05); this represents a 24% difference between the 2 IOL groups in combined visual acuity of 20/25 or better.

#### Binocular Distance-Corrected Defocus Testing at 3 Months

**Figure 4.**
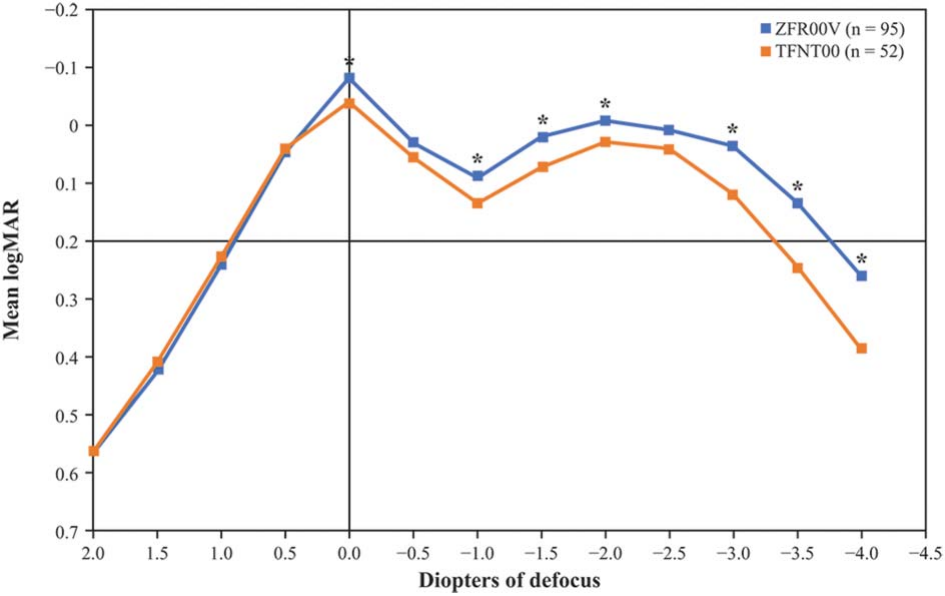
Binocular distance-corrected defocus curves for eyes implanted with the ZFR00V and TFNT00 IOLs at 3 months. *P ≤ .05 for ZFR00V vs TFNT00 IOLs.

Patients with ZFR00V IOLs had consistently better visual acuities at each defocus point than those with TFNT00 IOLs, with the ZFR00V IOL defocus curve demonstrating a higher peak acuity at 0.0 D defocus that extended further than that of the TFNT00 defocus curve, through −3.5 D (Figure 4). The ZFR00V IOL defocus curve was approximately 0.5 lines above that of TFNT00 IOL at 0.0 D (optical infinity), −1.5 D (optically simulating 66 cm viewing distance), and −2.5 D defocus (optically simulating 40 cm viewing distance) and was approximately 1 line better than the TFNT00 IOL at −3.0 D defocus (optically simulating 33 cm viewing distance), −3.5 D defocus (optically simulating 29 cm viewing distance), and −4.0 D defocus (optically simulating 25 cm viewing distance). The overall defocus range for 20/32 or better vision was approximately 0.4 D more with the ZFR00V IOL than with the TFNT00 IOL.

### Subjective Outcomes at 3 Months

Overall, nondirected patient reports of vision quality were excellent in the first and second eyes in both the ZFR00V and the TFNT00 groups, with no statistically significant differences between the IOL groups. Blurred vision/difficulty with vision of any severity was reported in 23 first eyes (24.2%) implanted with ZFR00V IOLs and 10 first eyes (19.2%) implanted with TFNT00 IOL. Furthermore, decreased vision was reported in 6 (6.3%) and 4 (7.7%) first eyes in the ZFR00V and TFNT00 IOL groups, respectively.

Visual symptom reports were similar in the first and second eyes for both IOL groups. The most frequently reported visual symptom was halos for both ZFR00V and TFNT00 IOL groups (42 [44.2%] vs 24 [46.2%] first eyes). Most reports of halos were of mild to moderate severity; severe halos were reported in 4 first eyes (4.2%) in the ZFR00V IOL group and 1 first eye (1.9%) in the TFNT00 IOL group. Other visual symptoms reported in both the ZFR00V and TFNT00 IOL groups included starbursts (5 [5.3%] vs 1 [1.9%] first eyes, respectively) and night glare (4 [4.2%] vs 1 [1.9%] first eyes, respectively).

### Safety

Medical complications/adverse events (AEs) were generally similar between the ZFR00V and TFNT00 IOLs first and second eyes at 3 months (Table 2). The most frequently reported medical complication/AE for the first eyes in the ZFR00V and TFNT00 IOL groups were mild-to-moderate posterior capsular opacification (4 [4.3%] vs 4 [7.7%]; *P* > .05).

**Table 2.** Medical findings (≥5%) in the first and second eyes at 3 months

Adverse events	First eyes, n (%)		Second eyes, n (%)	
	ZFR00V IOL (n = 95)	TFNT00 IOL (n = 52)	ZFR00V IOL (n = 95)	TFNT00 IOL (n = 52)
Blepharitis/meibomianitis	7 (7.4)	6 (11.5)	6 (6.3)	6 (11.5)
Dry eye/SPK/epithelial erosion/tear film insufficiency	8 (8.4)	4 (7.7)	8 (8.4)	4 (7.7)
PCO	24 (25.3)	10 (19.2)	23 (24.2)	9 (17.3)
Trace	20 (21.1)	6 (11.5)	20 (21.1)	7 (13.5)
Mild	3 (3.2)	3 (5.8)	2 (2.1)	1 (1.9)
Moderate	1 (1.1)	1 (1.9)	1 (1.1)	1 (1.9)
PC striae/wrinkles	5 (5.3)	5 (9.6)	5 (5.3)	4 (7.7)
Trace	4 (4.2)	3 (5.8)	5 (5.3)	3 (5.8)
Mild	1 (1.1)	1 (1.9)	0 (0)	0 (0)
Moderate	0 (0)	1 (1.9)	0 (0)	1 (1.9)
PVD/floaters	4 (4.2)	4 (7.7)	3 (3.2)	3 (5.8)
RPE changes	2 (2.1)	3 (5.8)	2 (2.1)	1 (1.9)

PC = posterior capsular; PCO = posterior capsular opacification; PVD = posterior vitreous detachment; RPE = retinal pigment epithelium; SPK = superficial punctate keratopathy

Three ocular serious adverse events (SAEs) in 3 eyes of 2 patients (1 bilateral event) were reported in the ZFR00V IOL group postoperatively and included 2 instances of cystoid macular edema (both eyes of 1 patient) and 1 corneal edema event, all of which resolved by 3 months. One ocular device–related AE, a broken IOL haptic that was noticed during the cataract surgery, required an IOL exchange.

Among TFNT00 IOL-implanted eyes, 7 ocular SAEs in 5 eyes of 3 patients were reported and included 2 cystoid macular edema (both eyes of 1 patient), 2 anterior capsular phimosis requiring Nd:YAG intervention (both eyes of 1 patient), 2 residual refractive errors requiring refractive surgical intervention (both eyes of the same patient with phimosis), and 1 intraoperative zonular dialysis and vitreous displacement resulting in Nd:YAG intervention. All reported SAEs were in line with the known AEs for the TFNT00 IOL and resolved during the study, except capsular phimosis, which stabilized by 3 months, and the residual refractive errors, which were ongoing when the patient exited the study.

## DISCUSSION

This study demonstrated satisfactory overall visual acuity performance for the TECNIS Synergy IOL, model ZFR00V, at 3 months postoperatively. The mean binocular corrected distance visual acuities were significantly better for the ZFR00V IOL group than that for the TFNT00 IOL group at distance and near (40 cm and 33 cm) vision, including some assessments under photopic and mesopic conditions. Furthermore, a higher proportion of patients implanted with ZFR00V IOLs achieved good binocular distance-corrected visual acuities at the tested reading distances, with many patients demonstrating good visual acuities at any reading distance. These patients may best demonstrate the clinical benefit of the ZFR00V IOL in providing a good range of vision through near viewing distances. Visual acuity data aligned with the mean defocus curve measured for each IOL group, where the ZFR00V IOL defocus curve demonstrated a higher peak acuity at 0.0 D and vision that extended further than that of the TFNT00 IOL defocus curve between the defocus points of +0.0 D and −3.5 D over the manifest refraction. The longer range of vision of the ZFR00V IOL that was predicted by preclinical modeling has been supported by the results of this clinical study.^6^ Notably, the AcrySof material is associated with more chromatic aberration compared with other IOL materials, and with the additional chromatic aberration correction feature of the ZFR00V IOL, both factors contribute to the performance differences observed in this clinical study.^7^ In addition, a 3-month follow-up limits the assessment of long-term effects observed with AcrySof material, such as that of lens glistenings that have been shown to increase light scatter and may potentially reduce vision quality over time.^8,9^ Moreover, recent studies suggest that IOL glistening will continue to increase over time rather than plateau.^10,11^

Previously published studies of the ZFR00V IOL and other presbyopia-correcting IOLs support the CDVA findings of this study. A prospective observational study of the ZFR00V IOL in patients undergoing bilateral cataract surgery reported that the mean visual acuity was better than 0.10 logMAR between +0.50 D and −3.00 D and 0.30 logMAR within an interval of +1.00 D to −4.00 D at 3 months postoperatively, as demonstrated by the binocular defocus curve.^12^ Another prospective multicenter single-arm study in patients who underwent bilateral implantation with the TFNT00 IOL reported a mean ± SD binocular CDVA of −0.05 ± 0.07 logMAR at 3 months postoperatively.^13^ Other studies of the TFNT00 IOL reported similar binocular CDVA values at 3 and 6 months postoperatively.^14–16^

Currently, 83% of individuals in the United States aged 50 to 64 years use smartphones.^17^ Furthermore, patients aged 50 years and older generally use near and intermediate vision to keep up with technology (smartphones, computers, etc).^18^ Therefore, it is important that currently available IOLs provide vision improvement over these distances. In this study, combined visual acuity was determined to assess consistent vision performance at all distances because some patients with good near vision may lose far vision or vice versa. Results of the combined visual acuities showed that 20/25 or better binocular corrected visual acuity at all tested distances (far to near) was achieved by a significantly higher proportion of patients implanted with the ZFR00V IOL compared with that of the TFNT00 IOL. The mean corrected visual acuities in our study were similar to uncorrected visual acuities (averaging a 2 to 3 letter improvement over uncorrected at far and near). Corrected visual acuities in this study are indicative of the actual (uncorrected) visual experience of patients, as evidenced by the fact that most of the patients (>92%) in both IOL groups reported no spectacle wear for far distances. Therefore, all the visual needs of the modern cataract patient may be well-addressed with a ZFR00V IOL.

Defocus curve findings have been used as a metric for clinical performance of IOLs in previous studies.^19–21^ In this study, the ZFR00V IOL defocus curve was approximately 0.5 lines better than for the TFNT00 IOL at 0.0 D defocus, −1.5 D defocus, and −2.5 D defocus at 3 months postoperatively. Furthermore, the ZFR00V IOL had consistently better visual acuities at each defocus point than the TFNT00 IOL (*P* ≤ .05), and the overall defocus range for the ZFR00V IOL at 20/32 or better was approximately 0.4 D broader than that of the TFNT00 IOL. These results suggest that the ZFR00V IOL may provide patients with vision at their preferred reading distances, particularly in the ranges of 40 cm and 33 cm.

Although multifocal IOLs improve vision across a range of distances, these IOLs can be associated with halo, starburst, and glare.^22–25^ In this study, incidence of severe halos and other visual symptoms was generally similar between the ZFR00V and TFNT00 IOLs (≤5%) and in line with other published studies of multifocal IOLs.^26–28^ However, responses to questionnaires about photic phenomena may change after the first 6 months of surgery because these phenomena typically improve with time due to adaptation.^29^ Patient-reported visual symptoms were excellent in both IOL groups, and medical and lens findings during the study support similar safety profiles between the ZFR00V and TFNT00 IOLs.

The main limitation of this study was the effect of the COVID-19 pandemic on patient follow-up visits due to different restrictions in each country in which the study was conducted, which affected patient accountability and led to exclusion of 1 study site from this analysis. However, although 1 site was excluded because of the COVID-19 pandemic, the sample size for this analysis was sufficiently powered. Furthermore, the study was performed for a limited power range of IOLs (+14.0 to +26.0), which excludes the more extreme eye anatomies. In addition, the nature of nondirected reporting of visual symptoms limited our ability to determine whether reports of decreased vision, halos, or starbursts were present in patients with or without corrected vision. Finally, patients enrolled in this study had healthier eyes due to a controlled clinical trial setting, and surgeons who participated in the study were highly experienced, which may have resulted in better outcomes compared with a more generalized clinical setting. In addition, a 3-month follow-up limits the assessment of long-term effects, such as IOL glistening, posterior capsular contraction, and posterior capsular opacification.

In conclusion, the TECNIS Synergy IOL, model ZFR00V, showed an acceptable safety profile and a clear advantage over the AcrySof PanOptix Trifocal IOL in clinical performance by broadening the range of vision across different distances (particularly at near distance) in patients with bilateral cataracts who underwent lens removal surgery. These results support the clinical use of the TECNIS Synergy ZFR00V IOL in patients older than 50 years who desire improved intermediate and near vision for day-to-day activities, thus supporting their vision needs and preferences.

**WHAT WAS KNOWN**

- Among patients undergoing cataract surgery, the use of monofocal IOLs leads to spectacle dependence for near vision, while multifocal IOLs are generally associated with limited intermediate vision and nighttime dysphotopsia.
- The new TECNIS Synergy IOL, model ZFR00V, combines diffractive bifocal technologies from the TECNIS multifocal and Symfony extended range-of-vision IOLs to provide improved distance vision in patients undergoing cataract surgery along with a continuous range of high-quality vision from intermediate through near vision.

**WHAT THIS PAPER ADDS**

- Results of this prospective, bilateral, randomized, comparative, multicenter study demonstrated better clinical performance particularly at near distances with TECNIS Synergy IOL, model ZFR00V, vs AcrySof PanOptix Trifocal IOL, model TFNT00, after bilateral cataract surgery.
- The ZFR00V IOL also showed a good safety profile and performance based on this global, multicenter assessment in which it was compared with a trifocal IOL.
